# Assessing False-Belief Understanding in Children with Autism Using a Computer Application: A Pilot Study

**DOI:** 10.1007/s10936-018-9579-2

**Published:** 2018-03-26

**Authors:** Emilia Carlsson, Carmela Miniscalco, Christopher Gillberg, Jakob Åsberg Johnels

**Affiliations:** 1 Institute of Neuroscience and Physiology, Speech and Language Pathology Unit, University of Gothenburg, Box 452, 405 30 Gothenburg, Sweden; 2 Institute of Neuroscience and Physiology, Gillberg Neuropsychiatry Centre, University of Gothenburg, Kungsgatan 12, 411 19 Gothenburg, Sweden

**Keywords:** Autism spectrum disorders, Theory of mind, False-belief understanding, School-age children, Computer application

## Abstract

We have developed a False-Belief (FB) understanding task for use on a computer tablet, trying to assess FB understanding in a less social way. It is based on classical FB protocols, and additionally includes a manipulation of language in an attempt to explore the facilitating effect of linguistic support during FB processing. Specifically, the FB task was presented in three auditory conditions: narrative, silent, and interference. The task was assumed to shed new light on the FB difficulties often observed in Autism Spectrum Disorder (ASD). Sixty-eight children with ASD (M = 7.5 years) and an age matched comparison group with 98 typically developing (TD) children were assessed with the FB task. The children with ASD did not perform above chance level in any condition, and significant differences in success rates were found between the groups in two conditions (silent and narrative), with TD children performing better. We discuss implications, limitations, and further developments.

**Electronic supplementary material:**

The online version of this article (10.1007/s10936-018-9579-2) contains supplementary material, which is available to authorized users.

## Introduction

Theory of mind (ToM) refers to the socio-cognitive capacity to understand that other people think and behave based on mental states (Premack and Woodruff 1978). ToM ability has implications for many aspects of children’s functioning, including social competence (Astington and Jenkins 1995; Astington 2003), peer acceptance (Dunn 1996; Dunn and Cutting 1999), as well as pragmatic language skills and early success in school (Astington and Pelletier 2005; Derks et al. 2016; Sutton 2003). Therefore, ToM has been investigated thoroughly in recent decades (Wimmer and Perner 1983; Baron-Cohen et al. 1985; Carlson et al. 2013) in both clinical groups (e.g., Meristo et al. 2012; Loukusa et al. 2014) and typically developing children (TD) (e.g., de Villiers and Pyers 2002; Dunn et al. 1991).

One frequently used way of testing ToM is based on the logic of location change and false-belief (FB) understanding, which was developed by Wimmer and Perner (1983) and further refined by Baron-Cohen et al. (1985). In the well-known “Sally–Anne” task, separate belief states of two characters are presented with respect to a hidden object. One of the characters (Sally) places a marble in a basket and then leaves the room. Then, the other character (Anne) comes in and moves the marble to a box. As Sally returns, the child is asked, “Where will Sally look for her marble?” If the child understands that Sally’s actions are guided by her *belief states* of the world, he/she will recognize that Sally holds a false belief in this case and that she therefore thinks that the marble is where she left it. Consequently, if the child points at the marble’s actual location in the box, he/she fails the task. This task has been given in a range of formats (mostly with picture or play material) to different populations, including typically developing children (Dunn et al. 1991; Lohmann and Tomasello 2003), children with ASD (Happé 1995), children with Williams syndrome (Tager-Flusberg et al. 1998; Van Herwegen et al. 2013), deaf children (Meristo et al. 2012), and children with language disorders (Nilsson and López 2016), since it was first introduced (e.g., Baron-Cohen et al. 1985). In addition to the FB question, the child is typically asked one or more control questions (e.g., “Where is the marble really?”). Control questions make interpretation of test question responses more reliable, since they help ensure that incorrect responses are not merely due to memory or general comprehension limitations.

A meta-analysis by Wellman et al. (2001) demonstrated that most TD children pass explicit FB tasks around the age of 4 years. Nevertheless, not all children pass the tasks in test situations, and some pass the tasks only when they are substantially older. The early study of Baron-Cohen et al. (1985) reported a clear difference between TD children and children with ASD. Indeed, the majority of children with ASD did not correctly predict Sally’s response, suggesting that they might not understand the belief states of others. There is now a large literature confirming that ToM is fundamentally difficult for many children with ASD, and that impairments in ToM may contribute to many of the social-communicative difficulties defining ASD (e.g., Frith 1989). Today, ToM is often routinely assessed in clinical practice during autism diagnostic evaluations, but the best way to assess ToM and FB has been the topic of much discussion.

A recent study of children with communication disorders investigated the interactional mechanisms involved in the administration of the Sally–Anne task (Korkiakangas et al. 2016). The authors concluded that it is often difficult to separate the child’s performance on the FB-task from other abilities such as sensitivity to interactional nuances/signals given by the test leader, but also that the interactional skills of the test person him/herself affect the results. The same conclusion—that assessment of FB is inherently socio-communicatively challenging—was also drawn in a study on TD children by Mauritzson and Säljö (2001). This issue might be particularly important to consider when working with children with ASD. Chevallier et al. (2014) explored differences in performance on a social cognition task depending on whether it was administered by a person or a computer and found that children with ASD and TD peers exhibited differing responses to interaction with a test leader. More precisely, while the ASD group and the TD comparison group performed similarly on the computerized task, the TD group outperformed the ASD group when the task was given in the presence of a test leader. This result suggests that children with ASD might underperform in relative terms during ordinary person-administered testing of social cognition (Chevallier et al. 2014).

In the current study, we evaluated the FB capabilities in children with and without ASD using a newly developed interactive computer application. We also aimed to explore the role of language in performing the FB task. Many studies have investigated the relationship between language ability and FB, and it has repeatedly been shown that FB performance is linked to language ability as assessed with various language tests (de Villiers 2007; de Villiers and Pyers 2002; Lohmann and Tomasello 2003; Milligan et al. 2007) in both TD children and clinical groups. The strength of the association may vary depending on how FB is measured and which aspects of language ability that is in focus. Indeed, there is no consensus regarding the eventual causal relation between language and ToM (Miller 2006). One view is represented by Olson (1989) who suggested that the development of ToM critically require a language to talk about the mind, a meta-language lexicon based on mental verbs such as “knows” and “thinks” (Olson 1989). Others have argued that syntactic language abilities, especially processing of sentence complements opens up necessary representational space for FB attribution (de Villiers and de Villiers 2000; de Villiers and Pyers 2002). More generally, in studies of deaf children, it has been shown that early exposure of language works as a critical scaffolding for FB understanding since deaf children born to hearing parents shows significant delays in ToM development, unlike deaf children born to deaf parents who jointly use sign language (Schick et al. 2007; Meristo et al. 2012). Apparently conflicting the critical role of language in FB development, studies using implicit measures, e.g., eye-tracking experiments, have shown that preverbal infants are sensitive to the beliefs of others (Onishi and Baillargeon 2005; Surian et al. 2007) and to FB during their second year of life (Baillargeon et al. 2010; Scott and Baillargeon 2009). However, much remains to be learned about the continuities between these early understandings of other minds, and the explicit form of FB that typically manifests around the age of 4 years in TD children (Perner 2014; Grosse et al. 2017).

Interestingly, while children with ASD are generally considered to have difficulties passing FB tasks, those with stronger language skills tend to perform better (Happé 1995). It has also been hypothesized that people with ASD who show good performance “solve ToM tasks in an unusually conscious and logical way” and do so “in a verbally mediated fashion” (Happé 1995, p. 852). This suggests that individuals with ASD with strong language skills might rely on language-mediated reasoning during belief attribution (Shield et al. 2016). In contrast, people without ASD might by default rely on non-verbal, intuitive processes during FB scenarios (Hill and Frith 2003; Happé 1995). Recent eye-tracking studies of FB tasks have revealed that TD individuals show a gaze orientation toward the location that indicates FB understanding, while at group level, individuals with ASD lack this ability even if some of them pass FB tasks (Senju et al. 2009).

Although these novel results are intriguing, there is a long way to go before eye-tracking methods can be transferred into the everyday work at clinics. An alternative approach is captured in the idea that FB is best measured not by performance in a single condition, but by the specific pattern of performance across conditions manipulating the availability of linguistic information. Guided by this logic, Forgeot d’Arc and Ramus (2011) introduced a verbal shadowing technique (through expressive verbal repetition) where TD adults performed FB tasks. The authors found that while linguistic interference decreased overall inference-making ability, it had no specific effect on explicit belief attribution. This result suggests that, in TD adults, FB performance is not affected any more by linguistic reasoning than is other types of cognitive reasoning.

Inspired by this line of work, the current study included a manipulation of the FB task, which was presented in three different conditions in order to explore the facilitating effect of language support in FB performance in groups with and without ASD. The three auditory conditions were as follows: (1) a verbal description of what is happening in the film (*narrative*), (2) a silent condition (*silent*), and (3) a verbal vocabulary interference condition (*interference*), i.e., the FB scenario was presented with random auditory interfering items intended to potentially hinder the use of language mediation during the processing of the FB scenario. We did not use the same verbal shadowing that Forgeot d’Arc and Ramus (2011) used with adults (i.e., expressive verbal repetition), since piloting suggested that this technique would be too difficult for children, especially for those with ASD.

The following three overarching research questions were addressed:

1. Can children with and without ASD complete the FB task using a computer tablet?
2. Do the children perform above chance level on the FB task in all three conditions?
3. In each of the three conditions, does the performance differ between the children with and without ASD? Related to this, we also specifically asked whether FB performance was verbally mediated in the ASD group such that performance limitations were particularly prominent in the interference condition.

## Methods

### Participants

Sixty-eight children (14 girls, 54 boys) from Gothenburg, Sweden, with an ASD diagnosis participated (mean age 7.5 years; range 5.9–9.1). They were recruited from a large longitudinal population study (*AUtism Detection and Intervention in Early life*, i.e., the AUDIE project) at the Child Neuropsychiatry Clinic (CNC), a clinic specializing in neuropsychiatric/neurodevelopmental assessments in Gothenburg, Sweden (Kantzer et al. 2013, 2016). The AUDIE cohort consisted of children who had screened positive for ASD as toddlers (N = 129), out of the children that were screened positive the parents of *n *=107 children gave their consent to be a part of the current research project. As toddlers they were assessed by an experienced multi-disciplinary team consisting of a child and adolescent psychiatrist, a neuropsychologist, a speech-language pathologist with many years of experience of working with children with ASD and other neurodevelopmental disorders at the clinic. There were *n *= 85 who were followed up at a mean age of 7.5 years. Note that not all children who had screened positive received an ASD diagnosis later. The present study reports data only for participating children who had a formal ASD diagnosis according to clinical consensus based on all available information from all professionals (*n *= 71). In addition, another three children were never administered the app due to experimenter or technical errors, which left a total of 68 children with ASD.

Ninety-eight children (48 girls, 50 boys) constituted an age-matched comparison group of TD peers with a mean age of 7.5 (range 5.2–9.0) years. The inclusion criteria used for the comparison group were: age above 5 years (since FB is generally acquired by then; e.g., Wellman et al. 2001), a standard score of at least 70 on the *Test for Reception of Grammar,* version 2 (TROG-2) (Bishop 2003, Swedish version 2009), and no reported ASD diagnosis. Participants in the comparison group attended three different elementary schools in western Sweden. Participant characteristics are reported in Table 1.

**Table 1:** Background data on all participants who completed the FB task

	ASD	Comparison group
	n = 52 M (SD)	n = 98 M (SD)
Age (years.months)	7.6 (0.9)	7.5 (0.9)
TROG-2 (standard score M = 100, SD = 15)	72.0 (20.1)	96.4 (13.6)
TROG-2 (block score, max 20)	7.2 (5.9)	13.4 (3.5)
CELF-4 Recalling sentences (raw score, max 70)	16.8 (16.5)	35.8 (9.5)^b^
CELF-4 Recalling sentences (scale score, max 20)	5.5 (5.2)	13.5 (2.9)
WASI matrices (t-score M = 50, SD = 10)	42.6 (13.3)	n.p^a^
ASSQ (max 54, cut off ASD > 18)	20.5 (8.8)	n.p.
ADOS severity score tot (max 10, cut-off ASD ≥ 4)	5.4 (2.4)	n.p.

Descriptive data is described for the children with ASD (n = 52) and the comparison group (n = 98)

^a^Not performed

^b^*n *= 97

### Material and Procedures

The data collection included a number of different child- and parent-rated measures/methods as described below. Children in the ASD group were assessed during one to two visits at the clinic, with each visit lasting approximately 60 min. The age-matched comparison group was recruited through their schools. Written informed consent was gathered from parents to all children that participated in the study. Children in the comparison group were assessed in a separate, quiet room at school and the assessment lasted approximately 45–60 min with breaks taken as needed.

#### False-Belief Understanding Task

**Fig. 1:**
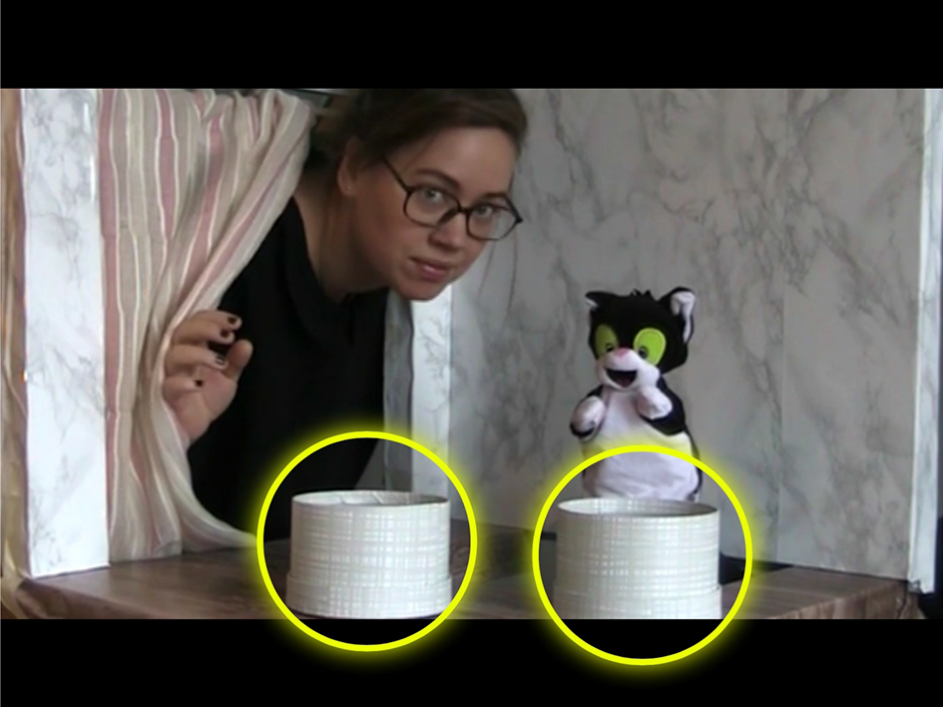
A screen dump from the FB task with “Johanna” and “Jansson the Cat”. The child answers the questions (i.e., “Where will Johanna look for the ball?” and “Where is the ball?”) by pointing at one of the yellow circles on the touch screen

An application on an interactive computer tablet was developed within the present study to explore first-order FB understanding based on change of location, using the classic Sally–Anne test as a model (Baron-Cohen et al. 1985). Children were verbally instructed to watch and then answer questions about short films. Within the self-instructing intuitive application, there is a film clip of a modified Sally–Anne-like scenario featuring two characters: a woman (“Johanna”) and a hand puppet (“Jansson the Cat”) (both well-known names to Swedish-speaking children) (see supplementary files). In the beginning of the clip, the child is verbally introduced to Johanna and Jansson the Cat. Jansson the Cat moves a ball from the box that Johanna had put it in while Johanna while Johanna is temporarily away. At the end of each trial, the child listens to questions within the application and responds by pointing (on the touch screen) to one of two yellow circles drawn around the two options (see Fig. 1). Two questions are asked: the FB question “*Where will Johanna look for the ball?*” and the control question “*Where is the ball really?*” The ball systematically changes places from left to right.

In order to explore the facilitating effect of verbal support in FB task performance, the 25-second film clip was shown in three different auditory conditions: (1) *narrative*, (2) *silent*, and (3) *interference.* In the *narrative* condition, the clip included a verbal description of the scenario given by a speaker voice within the application (1.4 words/s). In the *silent* condition, the clip was totally silent except for the questions at the end. In the *interference* condition, loud interfering auditory words (1.8 words/s), such as “ball,” “box,” “cat,” “Johanna,” and “Jansson,” were presented repeatedly in pseudo-random order. Two presentations of each condition were given in random order (randomized within the application), and the location of the ball was counterbalanced between presentations, i.e., in each of the three conditions one presentation had the ball placed in the left box and one presentation had the ball placed in the right box. The counterbalance of the locations demanded full attention to each presentation (rather than relying on answers to previous presentations). Two successful trials per condition were necessary for passing, which corresponds to a chance level of 25%. Each child needed about 3–4 min to complete the FB task. The child’s responses were registered and saved within the application automatically. Further information about the application can be obtained from the authors by request.

### Background Information

#### Syntactic Ability

The TROG-2 test was administered to measure receptive syntactic ability (Bishop 2003, Swedish version 2009). TROG-2 requires the child to match orally presented sentences of increasing syntactic difficulty with one of four pictures. The results are presented both in raw scores (number of correctly solved blocks out of a maximum of 20) and standard scores (M = 100, SD = 15 based on Swedish norms). The recalling sentences subtest of the Clinical Evaluation of Language Fundamentals–4 (CELF 4) was administered to measure expressive syntactic ability (Semel et al. 2003; Swedish version 2013). This test consists of 24 sentences and the child is asked to immediately recall each sentence produced by the examiner. Each item is scored from 0 (> 4 errors) to 3 (no errors) with a maximum score of 72. Here, the results are presented both in raw scores and scaled scores (M = 10, SD = 3; based on Swedish norms). Results are available for 66/68 children with ASD (missing data are due to children not cooperating during testing) and 97/98 children in the comparison group (one missing due to administration error).

#### Non-verbal Cognitive Ability

The matrices subtest of Wechsler Abbreviated Scales of Intelligence (WASI) was used as a measure of non-verbal cognitive ability (Wechsler 1999). Results are expressed in T-scores (M = 50, SD = 10) based on U.S. norms (no Swedish norms are available).

#### Autism Assessment

The Autism Diagnostic Observation Schedule-Generic (ADOS-G) (Lord et al. 2000) is a standardized, semi-structured play-based tool used for assessing communication, reciprocal social interaction, play, and behavior. Results were available for 50/52 children in the ASD group, with missing data being due to administration errors. ADOS severity scores were collected from the children’s medical records (collected at a mean age of 5 years); either module 1 or 2 was administered (depending on the expressive language level of the child). Raw scores were transformed to calibrated severity scores, with a maximum score of 10 and a suggested cutoff for ASD at ≥ 4 (Hus et al. 2014).

The Autism Spectrum Screening Questionnaire (ASSQ) parent version was completed at the time of FB testing for all participants in the ASD group. ASSQ is a parent report measure used to measure autistic symptomatology (Ehlers et al. 1999). The questionnaire contains 27 items with a three-level Likert scale. The test–retest reliability is reported to be very high for parent reports (*r *=.96), and validity was established by Ehlers et al. (1999) and by Posserud et al. (2009) by showing a clear correspondence between total ASSQ score and clinical diagnoses of ASD. A cutoff for ASD of > 18 has been suggested (Ehlers et al. 1999).

### Ethics

The study received ethical approval from the regional ethical review board in Gothenburg, Sweden (Case no. 723-13). All the parents provided written informed consent.

## Results

### Completion Rates on the FB Task

Of the 68 recruited children with ASD, a minority (*n* = 16/68; 25%) never managed to complete the full FB task. The reason for them not completing the task was often unclear to the test leader; it could reflect that they did not understand the task or did not want to participate. Independent *t* tests were used to compare the 16 participants who did not complete the full testing session with the remaining 52 children with ASD who did complete the FB task on all background variables. There were differences between these groups for sentence recall (CELF-4), (*t* (64) = 2.74, *p *=0.04) and a non-significant trend for the matrices subtest (WASI), (*t* (63) = 1.96, *p* = 0.07), suggesting that children who completed the FB task had higher expressive syntactic ability and non-verbal cognitive ability than those who did not complete the test. All 98 children in the comparison group managed to complete the FB task. In the remainder of the results, we focus, firstly, on the 52 children with ASD who completed the task; see Table 1.

### Success Rates for the FB Task (In Three Conditions)

**Fig. 2:**
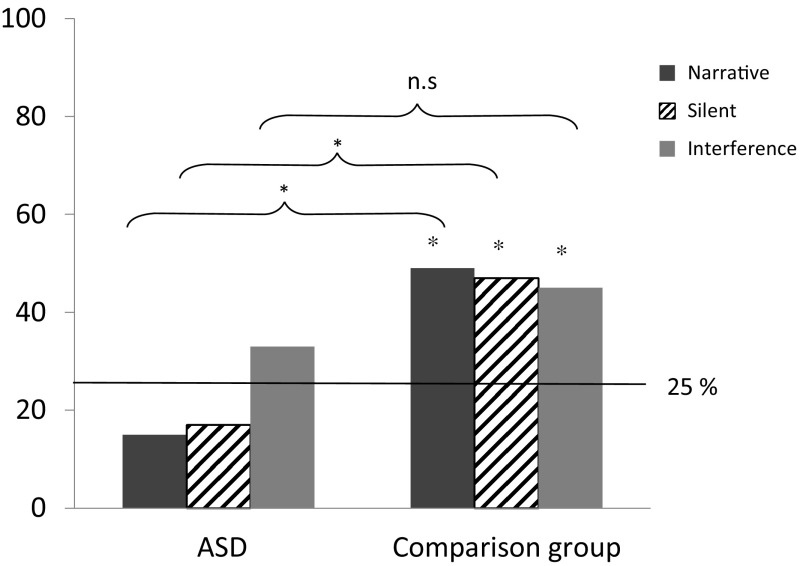
Success rates (percentage of the children passing) the FB task in the three conditions (*narrative*, *silent*, and *interference*) for the ASD group and the comparison group. Asterisk shows result significantly above chance level, *p* < .05. The claimers shows the comparison between groups. *n.s.* not significant

The probability of getting a fully correct answer by chance (i.e., both FB questions answered correctly) is 25%. To analyze the children’s performance in relation to the chance level, a binomial test was used with an alpha value of 0.05. The result shows that the ASD group did not perform significantly above chance level in any of the conditions for the FB questions. The success rates for the ASD group were 8 out of 52 in the *narrative* condition (15%) (*p *=0.96, binomial test), 9 out of 52 in the *silent* condition (17%) (*p *=0.93, binomial test), and 17 out of 52 in the *interference* condition (33%) (*p *=0.13, binomial test); see Fig. 2. However, the children with ASD performed above chance level for all control questions: 31 out of 52 passed the task in the *narrative* condition (success rate 60%) (*p *= < .001, binomial test), 25 out of 52 passed the task in the *silent* condition (48%) (*p *≤ .001, binomial test), and 26 out of 52 passed the task in the *interference* condition (50%) (*p *≤ .001, binomial test). In contrast, the comparison group (*n *= 98) performed above chance level on all FB questions in all conditions: 48 out of 98 passed the task in the *narrative* condition (success rate 49%) (*p *≤ .001, binomial test), 47 out of 98 passed the task in the *silent* condition (48%), and 45 out of 98 passed the task in the *interference* condition (46%). With regard to control questions, 84 out of 98 passed the task in the *narrative* condition (86%) (*p *≤ .001, binomial test), 82 out of 98 passed the task in the *silent* condition (84%) (*p *≤ .001, binomial test), and 70 out of 98 passed the task in the *interference* condition (71%) (*p *≤ .001, binomial test).

### Comparing the ASD and Comparison Group on the FB Task

To compare the performance of the participants with ASD who completed the FB task (*n *= 52) with the performance of those in the comparison group, Chi squared tests were performed. The results in the three different conditions are presented in terms of success rates. Statistically significant differences were seen between the groups in two of the conditions, i.e., in the *narrative* condition [χ ^2^ (1, *n *= 150) = 16.39, *p *< 0.001] and in the *silent* condition [χ^2^ (1, *n *= 150) = 13.64, *p *< 0.001], showing that children with ASD performed poorer than the comparison group on the FB question. No significant difference was found in the *interference* condition, although the trend pointed in the same direction [χ^2^ (1, *n *= 150) = 2.45, *p *= 0.12], see Fig. 2.

### Complementary Analyses of Language-Matched Subgroups

The ASD and comparison group were matched on chronological age but differed markedly on the language and cognitive measures. Perhaps most critically, on the TROG-2 test of receptive syntax, the ASD group scored nearly two standard deviations below the normative mean, whereas the comparison group scored close to normative levels. Since syntax appears to be the language skill that most consistently has been linked with ToM/FB performance (de Villiers 2005; de Villiers and Pyers 2002; Durrleman et al. 2016), we also performed complementary analyses by selecting participants from the ASD group with syntactic ability above standard score 70 (*n *= 22). The mean standard score on TROG-2 for this subsample of ASD participants was 92, which was not significantly different from that for the comparison group, *t* (118) = − 1.32, *p* = 0.21. When performing the same analyses as those presented above, a similar pattern was revealed in this subgroup: for the *narrative*, *silent*, and *interference* conditions, 6 out of 22 (27%; *p *= 0.5, binomial test), 6 out of 22 (27%; *p *= 0.5, binomial test), 10 out of 22 (48%; *p *= .024, binomial test) passed the test. Thus, the performance in the interference condition was significantly above chance level, but this was again not the case for neither the narrative nor the silent conditions. The ASD children with average syntactic ability performed above chance level on all control questions.

When comparing the children with ASD and average syntactic ability (*n *=22) with the comparison group, performance on the FB test was still numerically lower in the ASD group, but fell just shy of significance in both the *narrative* condition [χ^2^ (1, *n *= 120) = 3.42, *p *= 0.06] and the *silent* condition [χ^2^ (1, *n *= 120) = 3.12, *p *= 0.08]. Again, no between-group differences in performance could be seen in the *interference* condition [χ ^2^ (1, *n *= 120) = 0.12, *p *= 0.73].

## Discussion

Here, we have developed and initially evaluated the usefulness of a novel intuitive (self-instructing) FB task for an interactive computer tablet. Overall, our results show that young school-aged children with ASD have difficulties passing the FB task, as evidenced by the lack of above chance-level performance on the FB questions even though many understood the task in terms of where the object was physically located, reflected by the fact that they performed above chance level for the control question. This result fits with a large body of research pointing to a ToM deficit being characteristic of ASD (Dunn et al. 1991; Happé, 1995, 2015; Hill and Frith 2003; Loukusa et al. 2014). Our results show a largely similar pattern when complementary analyses were carried out in a subsample of the participants with ASD and age-average syntactic ability.

The current study was also novel in that, in an attempt to explore the role/facilitating effect of verbal reasoning in children with and without ASD, the FB task included a manipulation of language in an attempt to explore the facilitating effect of linguistic support during FB processing. The FB scenario was presented in three different auditory conditions: *narrative, silent*, and *interferenc*e. The *interference* condition was constructed with the aim to make it harder for the participants to use a language-mediated “hack-up” strategy when processing the FB scenario (Forgeot d’Arc and Ramus 2011), and it was hypothesized that this condition would reveal ASD-specific difficulties on the FB task even clearer than the other conditions, especially among participants with relatively strong language skills. This was, however, not the case. Finally, the results show that the comparison group, although performing above chance level, struggled with passing the FB task, even though the participants were 5 years or older. The results and their implications for clinical practice and future research is discussed below.

All participating children with ASD were recruited from a population-based screening cohort of toddlers (Kantzer et al. 2013, 2016). Hence, our study included the full range of abilities seen in ASD, including lower functioning children, a group often not included in research literature (Tager-Flusberg 1999). In our study, we found that the majority (75%) of the children with ASD completed the FB task (i.e., participated in the test, irrespective of whether they succeeded or failed). We observed quite a wide range of abilities (verbal and non-verbal) in both the group of children that completed the task and those who did not. Nonetheless, when comparing children who did versus did not manage to complete the full testing session (*n *= 16), significant differences were found in expressive syntactic ability as measured by the recalling sentences subtest (CELF-4), together with a non-significant trend in terms of lower non-verbal cognitive ability for those who did not complete the task. This suggests that although 52 children (a relatively large proportion) were able to complete the task, completion seems to require a certain level of intellectual functioning.

In the present study, we included children over the age of 5 years, since FB understanding is considered to be developed by age four in typically developed children (Dunn et al. 1991; Wellman et al. 2001). Unexpectedly, our results show that only 49% of the children in the comparison group passed the FB questions in the *narrative* condition, which was constructed to be similar to an ordinary FB task. This finding suggests that our application in its current form has limited clinical value and may be too difficult for children of this age; further studies are needed to better understand the underlying reasons, which include exploring performance on the task in older TD children, and in adults. In addition, the “app” could possibly be improved in certain ways for example one alternative could be to include a set of familiarization questions in the beginning.

Had the data shown that the ASD group performed above chance level on the task, it would have been meaningful to go on with further within-group analyses, i.e., comparing those who passed versus failed the FB-task. But since this was not the case, we opted not to. However, within-group comparisons for the comparison group have been performed, and are reported in the supplementary files; the results show that TD participant FB task passers tend to be older and more verbally skilled than TD participants who failed the task. This result is aligns with a large literature on FB understanding in the general child population, thus lending support for the usefulness of the application.

Still, a pivotal point in discussions of FB tasks in general has to do with construct validity (Bloom and German 2000), since classic FB tasks often rely on verbal eliciting and a social context of testing. In recent years, increased research interest has been devoted to “implicit” FB understanding assessed for instance by means of eye-tracking in infants (Onishi and Baillargeon 2005). This research provides a challenge to traditional accounts of FB development, since even preverbal infants appears to be sensitive to the belief states of others if the experiments are presented without the need for linguistic comprehension and overt answering (e.g., Rubio-Fernández and Geurts 2013). However, also the interpretation of performance differences on such implicit FB tasks is controversial (Perner 2014; Grosse Wiesmann et al. 2017). According to Ruffman (2014), there are important qualitative differences in the explicit and the implicit FB tasks, and performance of the latter can in many instances be explained by statistical learning of behaviors rather than necessarily by FB attribution. Also, a recent study by Grosse Wiesmann et al. (2017) showed that implicit and explicit FB tasks did not correlate in 3 to 4-years olds. Moreover, while the explicit FB tasks correlated with syntax and executive functioning, the implicit FB tasks did not (Grosse Wiesmann et al. 2017). The current FB application, it appears to be somewhere in-between a classic socially and linguistically administered FB task and an implicit task since it in part includes a linguistic framing of the scenario (including control questions). Such a visual presentation allows the child to potentially keep track of a protagonist’s perspective over a course of events, without linguistic demands being critical and potentially disrupting the perspective-tracking process.

Worth noting, also, Dumontheil et al. (2010) used a computerized test of ToM-related communication skills in five age groups (7–27 years) and found that the performance on the task increased with age, indicating that ToM functions continues to develop until late adolescence, potentially in parallel with developments in frontally mediated executive functions (Dumontheil et al. 2010). It is possible that the use of a tablet or computer when performing the FB task requires more from the child’s executive functions than an ordinary FB test situation, which should be evaluated in future research. Conversely, the low success rate in the present study could possibly also be attributed to the social environment in which the test was administered. For example, it has been shown in previous studies that the test leader can have an impact on the performance of FB/ToM-tasks (Chevallier et al. 2014). We believe that future research studies are needed to better explain the associations between different types of FB tasks and which factors—including chronological age, language support/demands and executive demands—that effect performance differentially.

Most FB tasks are based on an explicit narration of the scenario including a verbal question, and this describes our FB task in the *narrative* condition. Yet, this may lead to a situation where certain populations with limited language abilities have difficulties grasping the test (Milligan et al. 2007). According to this logic, we carried out complementary analyses of the children in the ASD group with average syntactic ability (*n *= 22) and the comparison group. The pattern was largely unchanged, except for in the *interference* condition, where the children with ASD and average syntactic ability (unexpectedly) performed above chance level and not differently from the comparison group. This was contrary to our hypothesis, i.e., that group differences (favoring the TD group) would be clearest in the *interference* condition. This was an unexpected finding and should be interpreted with caution because of the small numbers and the overall low test performance. Still, it might be interesting to speculate about the reasons for this observation, since it could provide guidance for future research using this specific computer application, and more generally on social cognition in ASD. One potential explanation may be related to an unusual response to sensory and perceptual stimuli often seen in individuals with ASD (Billstedt et al. 2007; Leekam et al. 2007). The relatively strong performance in the *interference* condition could potentially reflect that the children with ASD got alerted in this condition, which perhaps made them focus more and therefore pass the FB task. This interpretation could be in line with a recent study by Kleberg et al. (2016) showing that children with ASD might have an atypical functioning of the phasic alerting system during social processing. Interestingly, Kleberg et al. (2016) showed that children with ASD performed slower than children in the comparison group on a social orienting task, but when a face stimulus was presented together with a spatially non-predictive sound stimulus, the pattern was reversed such that the ASD group performed relatively faster in this condition. It is thus presumed that increases in auditory alerting cues induce a state of readiness to respond, which partly normalizes socio-cognitive functioning (see also, O’Connell et al. 2006). Likewise, a few other relevant studies of priming in the form of verbal instructions (e.g., Begeer et al. 2006) or presentation of non-speech sounds (Whitehouse and Bishop 2008) have shown that these manipulations enhance attention to the target stimuli in children with ASD. Taken together, these previous studies indicate that the auditory interference condition in our study might have affected the results positively by inducing a state of readiness and alertness. Still, since our result was not predicted a priori, we believe the result should be considered tentative until further replication has been performed.

The present pilot study has limitations. First, it would have been valuable to include complementary testing of FB understanding in the classical person-administered way described by Baron-Cohen et al. (1985), and with eye-tracking-based measures of implicit ToM (e.g., Senju et al. 2009). With such additional FB data, it would have been possible to compare the results obtained in our experiment with the outcome of those tests in order to explore associations and dissociations in performance depending on how FB is measured. It would also have helped us compare computer- and person-mediated performance on FB tasks, similar to what Chevallier et al. (2014) did for another social cognitive task relating to the processing of intentions. Also, although we recruited a relatively large sample, it is nonetheless somewhat limited considering that the outcome data is categorical (pass vs. fail). Hence, potential power issues need to be remembered when interpreting the results of this pilot study. The design, particularly the independence of each trial, could also be discussed. Since every child saw the scenario in three different auditory conditions, it cannot be ruled out that the performance in one condition influenced that in the next. One possible way of handling this issue could have been to present different stories in each condition. On the other hand, one strength of our design is that every condition is the same and therefore comparable, and that the true placement of the ball to the left versus right was counterbalanced and presented in a random order.

## Summary

In conclusion, our results show that the young school-age children with ASD did not pass the FB task above chance level, whereas typically developing children did so. However, in certain aspects, the computer application did not seem to work as anticipated, since it turned out to be unexpectedly demanding even for the TD comparison group. This may have masked any influence of the manipulation of linguistic support during FB processing in the study group. The FB task used in the current study is novel since it was presented on an interactive computer tablet. At clinics, an interactive computer tablet application has the advantage of being fast and easy to administer. With additional revising and improvements, our computer application may serve as a standardized and less “social” way of testing FB, i.e., one that is not as dependent on the interaction between the examiner and the child.

## Electronic supplementary material

Below is the link to the electronic supplementary material.

Supplementary material 1 (DOCX 16 kb)
