# Supplementary material for: Assessing False-Belief Understanding in Children with Autism Using a Computer Application: A Pilot Study
Source: J Psycholinguist Res. 2018 Mar 26;47(5):1085–99. doi: 10.1007/s10936-018-9579-2 (PMC6153757; doi:10.1007/s10936-018-9579-2)
Supplement: Supplementary file 1 — Supplementary material 1 (DOCX 16 kb) [file 10936_2018_9579_MOESM1_ESM.docx]

**Complementary analyses in the comparison group**

One way-ANOVA between-subjects analyses were conducted within the comparison group, to compare those who failed the FB task with those who succeeded the task in the three conditions separately, i.e., the narrative condition, the silent condition, and the interference condition. The results show that those who passed the FB task (in all three conditions) were significantly older and had higher syntactic language ability, measured by both TROG- 2 and the recalling sentences subtest (CELF-4), than those who failed the task.

|  | **Narrative condition** | **Silent condition** | **Interference condition** |
| --- | --- | --- | --- |
| **Age** (years;months) | F (1, 96) = 7.20 *** | F (1, 96)=4.30 *** | F (1, 96) = 6.38 *** |
| **TROG-2** (standard score) | F(1, 96) = 11.79* | F(1, 96)=6.67 *** | F(1, 96) = 6.77, *** |
| **TROG-2** (block score,  max 20) | F(1, 96) = 18.25* | F(1, 96)=10.59 *** | F(1, 96) = 12.39 * |
| **CELF-4 Recalling**  **Sentences**  (raw score, max 70) | F(1, 95) = 16.75* | F(1, 95)=6.77 * | F(1, 95) = 14.77 * |
| **CELF-4 Recalling**  **Sentences**  (scale score, max 20) | F(1, 95) = 9.71* | F(1, 95) = 2.65 | F(1, 95) = 9.61 * |

* = *p* < .05
